# Behaviour and Movement Activity of Stallions and Geldings in Group Housing

**DOI:** 10.3390/vetsci13070660

**Published:** 2026-07-07

**Authors:** Rhoda C. Apitzsch, Sarah Handel, Konstanze Krueger

**Affiliations:** Faculty of Agriculture, Economics and Management, Nuertingen-Geislingen University, Neckarsteige 6-10, 72622 Nuertingen, Germany; r.apitzsch@mqs.de (R.C.A.); sarah@tssl-handel.de (S.H.)

**Keywords:** stallions, geldings, horse behaviour, group housing, social behaviour, movement activity

## Abstract

There has been little research on the social behaviour of stallions and geldings kept together in group housing systems. The aim of this study was to investigate differences in behaviour and movement activity between stallions and geldings kept together in group housing. Behavioural observations and GPS measurements were conducted in Germany from July to September 2024 in four mixed groups of horses in Germany. The groups comprised 35 animals, 18 stallions and 17 geldings. The horses were observed for three consecutive days over eight hours per day. No injuries or stereotypic behaviours were observed throughout the observation period. Stallions displayed significantly higher levels of affiliative, agonistic, dominance, ritualised, reproductive and play behaviours than geldings (all *p* < 0.05). However, attack, threat, comfort, avoidance, and resting behaviours, as well as movement activity did not differ significantly between stallions and geldings. Greater space availability per horse was associated with increased comfort, resting, reproductive and play behaviours, and dominance behaviour decreased and attacks tended to decrease with increasing space availability. The horses moved an average distance of 2.36 km during the observation periods.

## Simple Summary

Group housing is considered beneficial for horse welfare because it allows horses to express natural social behaviours. Nevertheless, stallions are still frequently housed individually due to concerns about aggression and injuries, as well as management challenges such as handling by humans, group integration and increased monitoring requirements. This study investigated the behaviour and movement activity of stallions and geldings kept together in group housing in Germany. Four groups of male horses, comprising 18 stallions and 17 geldings, were observed over three consecutive days for eight hours per day. GPS trackers were used to measure movement activity. No injuries or stereotypic behaviour were observed during the observation period. Stallions displayed significantly higher levels of affiliative, agonistic, dominance, ritualised, reproductive and play behaviours than geldings. However, attack behaviour and movement activity did not differ significantly between stallions and geldings. Horses kept in larger enclosures showed more comfort, resting and play behaviours and attack behaviour tended to decrease with increasing space availability. The results indicate that group housing of stallions and geldings is possible without increased injury risk under suitable management conditions and this may support species-appropriate social behaviour and horse welfare.

## 1. Introduction

The management of horses in a species-appropriate way is becoming more common in central Europe due to ethical considerations and growing societal awareness of animal welfare [1]. Horses are highly social animals and in nature live in stable social groups in which continuous social interaction is an essential part of their behaviour [2,3,4]. Group housing systems are therefore generally considered more species-appropriate than individual housing, as they allow horses to express species-specific behaviours, move freely and optimise social contact [2].

Despite these advantages, many domestic horses, particularly stallions, are still housed individually, often with limited opportunities for free movement and social contact with conspecifics [5]. This practice is largely driven by concerns about aggressive interactions and the associated risk of injuries [5,6]. However, restricted social contact has been associated with increased stress and the development of abnormal behaviours, highlighting the welfare limitations of individual housing systems [7,8,9,10]. Stereotypic behaviours are repetitive, invariant behaviour patterns to relieve stress and are widely regarded as indicators of compromised welfare in horses [11]. They have been linked to suboptimal management factors such as restricted forage availability, limited social contact and insufficient opportunities for movement [11].

Under natural conditions, horses form two main social group structures: harem groups and bachelor bands [12]. Harem groups usually consist of between one and five stallions, several mares, and their offspring [12,13], whereas bachelor bands are typically comprising young males that have left their natal groups and older stallions that have lost or not acquired a harem [12,13]. The members of these groups engage in frequent social interactions, including agonistic behaviours such as threats, avoidance, and fighting, which play an important role in establishing dominance hierarchies and the development of social behaviours [14,15,16]. In addition to agonistic interactions, affiliative behaviours such as mutual grooming, friendly approaches [3] and social play are central to the establishment and maintenance of social bonds and group cohesion [3,17,18].

However, findings on social behaviour in domestic horses, particularly in stallions, are not entirely consistent. Several studies indicate that stallions can be kept successfully in groups with relatively low levels of aggression under appropriate conditions [19,20]. In contrast, other studies report increased aggression during group formation or where space is limited [5,21]. These discrepancies highlight the role of various factors such as space availability, group composition and management practices in influencing social behaviours [4,5,21].

In addition, the influence of reproductive ability on social behaviour in male horses has been investigated [22]. Castration has been shown to affect certain behavioural patterns, with geldings exhibiting higher levels of affiliative behaviour than stallions [22]. However, castration did not affect the frequency of agonistic behaviours, and the level of aggressive behaviour remained unchanged after castration [22].

To date, there have been few studies analysing the social behaviour of stallions and geldings kept together in group housing. To investigate this topic, this study employed behavioural observations and GPS measurements of movement frequencies in four groups of mixed stallions and geldings. The aim was to investigate differences in social behaviour and movement activity between stallions and geldings in group housing. We asked: (a) Do injuries occur in mixed stallion—gelding groups? (b) Do stallions and geldings display stereotypic behaviour when kept in group housing? (c) Do stallions and geldings differ in the frequencies of displaying social behaviours, such as affiliative, agonistic, ritualised, reproductive, comfort, resting and play behaviour? (d) Do stallions and geldings differ in the distances they move within the group housing systems.

## 2. Materials and Methods

### 2.1. Ethics Statement

This study is registered under the number 2024_55_07.08.2024 at Nuertingen-Geislingen University. Permission was given from the Animal Welfare Board and the Ethics Board of Nuertingen-Geislingen University, as the present study was not considered an animal experiment and all claims of the Data Protection Directive of the European Union (DSGVO 2016) were fulfilled. The obtained data was transferred into anonymous, written form directly after conducting the material. From the anonymous raw data, no individual data of the participating observers was used in this study. The methods of the study were visualised in graphs without showing persons. The study published no pictures, videos or any data from which personal data could be drawn, either in the manuscript or in any Supplementary Material. Informed consent was obtained from all participating farm owners.

### 2.2. Observation Period and Locations

We observed 35 male horses (*N* = 35) in four mixed groups of stallions and geldings at four different equestrian facilities. The facilities were selected regardless of the breed of the horses, the group size or the group housing method. However, a few requirements were set to ensure that the facilities were comparable: (a) stallions and geldings had to be kept together in group housing, (b) each group was to include at least one adult gelding and at least two adult stallions, and (c) there had to be no visual contact with mares.

The first observation took place between 27 July 2024 and 29 July 2024 in Simmersfeld (Germany). The group comprised 24 horses (*n* = 24), including 11 stallions and 13 geldings, which were kept together in a paddock trail system. At the time of observation, the group had been unchanged for three months, with no horses being added or removed. Mares were located out of sight of the group. During the observation period, the horses had unrestricted access to hay via three covered hay racks, each filled with two large square hay bales. The hay was covered with hay nets to slow the feeding rate. Two straw-bedded shelters were available for weather protection and as a resting area. The horses had permanent access to grass in two pastures. The paddock trail area available was 7088 m^2^, and an additional 5255 m^2^ of pasture, giving a total area of 12,343 m^2^. The area per horse was therefore 514 m^2^.

The second observation took place from 30 July 2024 to 1 August 2024 (Table 1) in Opfenried ( Germany). The group comprised two adult stallions and two adult geldings (*n* = 4). For the past 10 years the horses had been kept in open stabling with separate resting, shelter and feeding areas and access to an outside paddock during the day. In addition to the paddock, the horses were allowed onto the adjacent pasture of 2878 m^2^ for a few hours at a time. The horses were housed in outside boxes at night. The stallions were kept individually, while the two geldings were kept together in one stable. There were no mares in the vicinity. Hay was fed at intervals throughout the day. The available area was 576 m^2^. Thus, a space of 144 m^2^ per horse was available during the day.

The third observation took place from 8 September 2024 to 10 September 2024 (Table 1) in Zeven ( Germany). The group comprised two adult and one three-year-old stallion and one adult gelding (*n* = 4). The group was housed in open stabling with separate resting, shelter and feeding areas and outside paddock. The group had been unchanged for three months. There were no mares in close proximity to the stallions. Hay was fed twice daily in hay racks. During the observation period, the horses did not have access to pasture. The housing area totalled 2364 m^2^, corresponding to 591 m^2^ per horse.

The fourth observation took place from 12 September 2024 to 14 September 2024 (Table 1) in Niederlauken ( Germany). The group had been together for two years and comprised two adult stallions and one adult gelding (*n* = 3). They were kept on pasture with no mares close by. The trees at the edge of the pasture provided shelter from the weather. The pasture covered an area of 4998 m^2^, corresponding to 1666 m^2^ per horse.

The observations took place over three consecutive days for each group. Data were collected for eight hours each day. This time frame was selected to ensure standardised, continuous data collection that was also logistically feasible. The times of the observation intervals were spread semi-randomly, to obtain a representative overview of the horses’ behaviour throughout the day [23]. Observations were carried out as follows:

**Table 1:** Schedule of observations.

Equestrian Facility	Date	Observation Time
1	27 July 2024	13:00 h–21:00 h
	28 July 2024	8:00 h–12:00 h; 14:00 h–18:00 h
	29 July 2024	8:00 h–12:00 h; 12:30 h–16:30 h
2	30 July 2024	12:00 h–16:00 h; 17:00 h–21:00 h
	31 July 2024	8:00 h–12:00 h; 14:00 h–18:00 h
	1 August 2024	8:00 h–12:00 h; 12:30 h–16:30 h
3	8 September 2024	11:30 h–17:00 h; 17:45 h–20:15 h
	9 September 2024	8:00 h–14:00 h; 15:00 h–17:00 h
	10 September 2024	8:00 h–12:30 h; 13:15 h–16:45 h
4	12 September 2024	9:45 h–14:00 h; 16:00 h–19:45 h
	13 September 2024	8:00 h–12:00 h; 14:00 h–18:00 h
	14 September 2024	8:00 h–12:00 h; 12:30 h–16:30 h

### 2.3. Animals

A total of 35 horses were observed (*N* = 35), comprising 18 stallions and 17 geldings of various breeds and ranging in age from 1 to 19 years (Table 2). The median age was 7 years (range: 1–17 years) for stallions and 15 years (range: 3–19 years) for geldings.

### 2.4. Experimenters and Their Tasks

Qualitative, continuous field observations [23] were conducted by two observers using direct observation. The observers were aware of the behavioural categories being recorded. For the large group, the observation area was spatially divided between the two observers, with each observer responsible for data collection in one half of the area. Each horse was therefore observed and recorded by only one observer responsible for the area in which they were located. When a horse moved from one area to the other, responsibility for its observation was transferred to the observer assigned to the new area. Both observers used the same predefined ethogram and observation protocol. For the three smaller groups, both observers simultaneously monitored the same animals. Any uncertainties regarding behavioural classification were discussed directly and resolved by mutual agreement. One observer was responsible for recording the observations.

To minimise any effect of the presence of the observers, the observations were carried out from outside the paddocks wherever possible. At two of the four facilities, this was not possible due to the size and shape of the areas so the observers were positioned inside the paddocks where they could oversee the entire area and all the horses. The behaviour of all horses within a group was recorded directly and continuously by observers in the field [23]. Behavioural observations were conducted without the use of technical recording devices such as cameras or automated monitoring systems, whereas movement activity was assessed separately using GPS devices. Observations were conducted for eight hours on three consecutive days and documented in a standardised handwritten format. The behaviours were categorised and numbered in advance, as described in Section 2.6.1.

In addition, the geographical location and weather conditions on the respective days were recorded for each facility, and age, castration status, breed and other relevant information was recorded for each horse.

### 2.5. Technical Equipment

Five Qstarz BT1000XT GPS trackers (Qstarz International Co., Ltd., Taipei, Taiwan) were used in the study. The units were preconfigured to record positional data at 5 s intervals. They recorded GPS-based movement data, including geographic coordinates, from which the distance travelled was calculated and expressed in kilometres. The trackers were secured inside a plastic bag, to protect them from dirt and moisture, and attached to a leather collar around the horses’ necks.

The GPS trackers remained attached to the horses for at least eight hours per day and were, in some cases, left on during observation breaks. At the end of each day, the devices were removed, the data were downloaded and cleared, and the units were recharged overnight before being reattached the following morning.

To correct potential inaccuracies in the GPS measurements, one of the five trackers was positioned as a control GPS device at the entrance to the horses’ paddock. The distances measured by the control GPS highlight errors caused by satellite jumps.

### 2.6. Observation Procedure

For this study, empirical data were collected, including behavioural observations and GPS data.

The horses were individually identified prior to the start of observations based on detailed descriptions provided by their owners, and each animal was visually confirmed on site. Following this identification process, there was an acclimatisation period to minimise potential observer effects. The observers spent approximately one hour in proximity to each group without engaging in any interactions, allowing the horses to become accustomed to their presence before systematic data collection began.

#### 2.6.1. Behavioural Categories

The behavioural categories were predefined and standardised prior to data collection [14,24,25] (see Table 3).

#### 2.6.2. GPS-Based Movement Recording

In the first group, movement data were recorded for four randomly selected horses (two adult stallions and two adult geldings), as only four GPS trackers were available and the group was too large to equip all horses. In the other three groups, movement data was recorded for all the group members. In total, movement data was recorded for over three days for each of 15 horses, nine stallions and six geldings.

The movement activity was recorded using GPS trackers for eight hours per day on three consecutive days per group, resulting in a total of 24 h of movement data per group, 96 h in total. The trackers were activated daily at the start of the observation period in the morning and deactivated again in the evening.

### 2.7. Data Collection and Analysis

#### 2.7.1. Data Preparation

All behavioural data and GPS data were recorded and subsequently transferred to Microsoft Excel. Additional variables, including age, housing system, facility, castration status and the available space per horse, were then added to the dataset. The GPS data had to be corrected because the trackers were either left on the horse or temporarily removed during observation breaks. To ensure an accurate analysis of the movement data, these break intervals were identified retrospectively and filtered out of the datasets. Furthermore, erroneous movement distances caused by satellite jumps, as identified using the stable control GPS, were subtracted from the movement data of the respective group. Subsequently, the corrected movement distance was divided by the actual operating time of the GPS tracker to calculate movement activity in kilometres per hour. To ensure comparability among individuals despite differences in recording duration, movement distances were then standardised to an eight-hour observation period.

Every evening, information on recording duration and distance travelled was extracted from the GPS trackers using QTravel software, version 1.55.003.

#### 2.7.2. Data Analysis

All data were analysed using statistical software ‘R’ (version 4.5.1) and the R package R Commander. As a large proportion of the collected data was not normally distributed (Shapiro–Wilk test: *p* < 0.05), non-parametric methods were used.

The Mann–Whitney U test was applied to analyse median differences in behaviour and GPS data between stallions and geldings. Generalised linear models (GLMs) were used for multivariate factor analyses. These models are suitable for investigating the relationship between a dependent variable and several independent variables in non-normally distributed data. Depending on the data structure, different distributions and link functions were specified.

The behavioural data were initially analysed using a Poisson model. However, this resulted in overdispersion [26]. The ratio of residual deviance to residual degrees of freedom was substantially greater than 1 [26]. In this case, it is recommended to set the family argument of glm() to quasipoisson (link = “log”) [26]. For the standardised GPS data, a Gamma distribution with a log link function was used in the respective GLM, as these data were also not normally distributed (Shapiro–Wilk test: *p* < 0.05). Binomial tests were used to examine frequency distributions of characteristics with two levels [27]. For all tests, the significance level was set at *p* < 0.05. A *p*-value between 0.05 and 0.10 was considered a statistical trend.

## 3. Results

### 3.1. Behaviours

Throughout the entire observation period, no stereotypic behaviour or injuries caused by interactions between horses were observed. Furthermore, we recorded affiliative, agonistic, attack, threatening, dominance, ritualised, reproductive, comfort, avoidance, resting and play behaviours and movement activity. An overview on the observed frequencies of behaviours in stallions and geldings is provided in Table 4.

#### 3.1.1. Affiliative and Agonistic Behaviour

**Figure 1:**
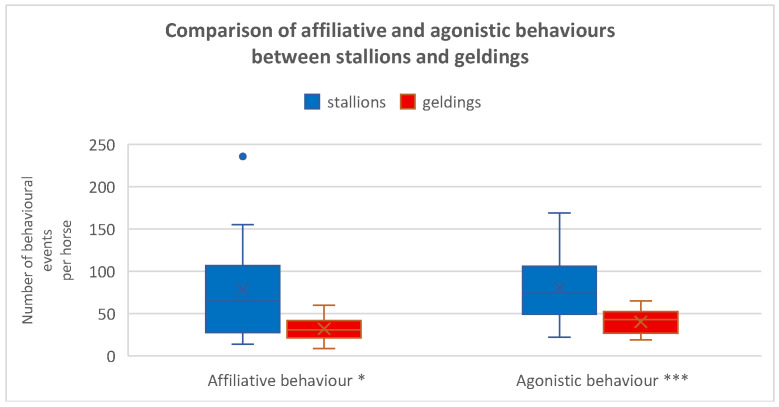
Stallions exhibited significantly higher levels of affiliative and agonistic behaviour than geldings (Mann–Whitney U test: all *p* < 0.05). *: *p* ≤ 0.05; ***: *p* ≤ 0.001.

Affiliative behaviours occurred significantly less frequently than agonistic behaviours (binomial test: *N* = 4109, *p* = 0.01; Figure 1; Table 4). Stallions exhibited significantly higher levels of both affiliative and agonistic behaviour than geldings (Mann–Whitney U test: affiliative: *N* = 35, W = 227, *p* = 0.02; agonistic: *N* = 35, W = 254.5, *p* < 0.001; Figure 1; Table 5). The available space per horse and the age of the horses had no effect on agonistic and affiliative behaviour (GLM: *N* = 35, all *p* > 0.05).

#### 3.1.2. Attack Behaviour

**Figure 2:**
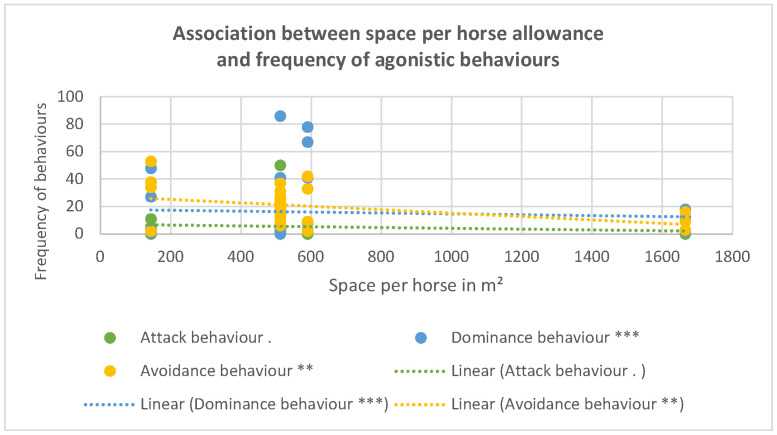
Association between space per horse and frequency of attack (GLM: *N* = 35, t = −1.85, *p* = 0.07), avoidance (GLM: *N* = 35, t = −2.77, *p* = 0.01) and dominance behaviours (GLM: *N* = 35, t = −3.64, *p* = 0.001); **: *p* ≤ 0.01; ***: *p* ≤ 0.001; the dotted trend lines represent a linear regression and illustrate the association between space per horse and these behaviours. A dot (.) indicates a trend.

There was no significant difference in attack behaviour between stallions and geldings (Mann–Whitney U test: N = 35, W = 174, *p* = 0.49; Table 4 and Table 5). The age of the horses had no significant effect on attack behaviour (GLM: N = 35, *p* > 0.05). There was a tendency for horses with less space per horse to show more attack behaviour; however, this effect was not statistically significant (GLM: N = 35, t = −1.85, *p* = 0.07; Figure 2).

#### 3.1.3. Threatening Behaviour

Threatening behaviour did not differ significantly between stallions and geldings, although there was a trend for median values to be higher in stallions (Mann–Whitney U test: *N* = 35, W = 205, *p* = 0.09; Table 4 and Table 5). Space availability per horse and dominance behaviour were positively associated with threatening behaviour. Greater space availability per horse was associated with higher levels of threatening behaviour (GLM: *N* = 35, t = 3.14, *p* = 0.004). Furthermore, horses displaying higher levels of dominance behaviour also showed threat behaviour more frequently (GLM: *N* = 35, t = 5.09, *p* < 0.001). The age of the horses and group size were not significantly associated with threatening behaviour (GLM: *N* = 35, all *p* > 0.05).

#### 3.1.4. Dominance Behaviour

**Figure 3:**
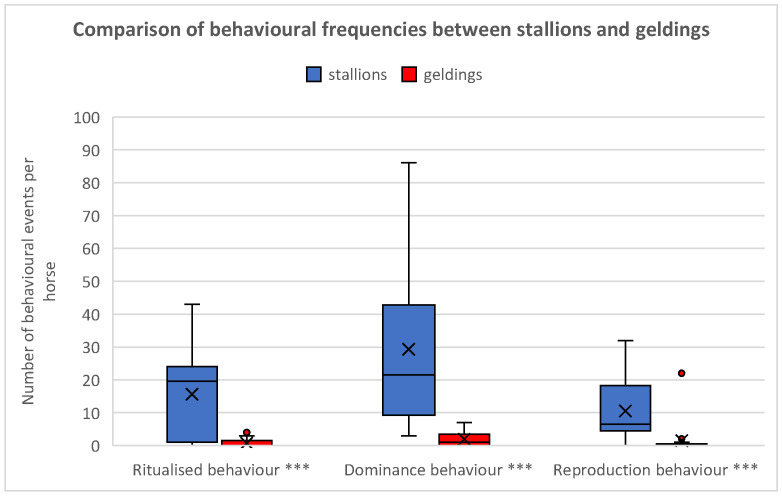
Stallions exhibited significantly higher levels of ritualised, dominance and reproductive behaviour than geldings (Mann–Whitney U test: all *p* < 0.001). ***: *p* ≤ 0.001.

Stallions exhibited significantly higher levels of dominance behaviour than geldings (Mann–Whitney U test: *N* = 35, W = 290, *p* < 0.001; Figure 3; Table 4 and Table 5). Age was positively associated with dominance behaviour, with older horses displaying higher levels of dominance behaviour (GLM: *N* = 35, t = 2.76, *p* = 0.01). Space availability per horse was negatively associated with dominance behaviour, indicating that horses with less space exhibited higher levels of dominance behaviour (GLM: *N* = 35, t = −3.64, *p* = 0.001; Figure 2). Furthermore, reproductive behaviour was positively associated with dominance behaviour, with horses showing higher levels of reproductive behaviour also displaying more dominance behaviour (GLM: *N* = 35, t = 4.39, *p* < 0.001).

#### 3.1.5. Ritualised Behaviour

Stallions exhibited significantly higher levels of ritualised behaviour than geldings (Mann–Whitney U test: *N* = 35, W = 261.5, *p* < 0.001; Figure 3; Table 4 and Table 5). Furthermore, age and reproductive behaviour were positively associated with ritualised behaviour. Older horses displayed higher levels of ritualised behaviour (GLM: *N* = 35, t = 3.47, *p* = 0.002). In addition, reproductive behaviour was positively associated with ritualised behaviour (GLM: *N* = 35, t = 2.53, *p* = 0.03). Space availability per horse was not significantly associated with ritualised behaviour (GLM: *N* = 35, t = −0.13, *p* = 0.90).

#### 3.1.6. Reproductive Behaviour

**Figure 4:**
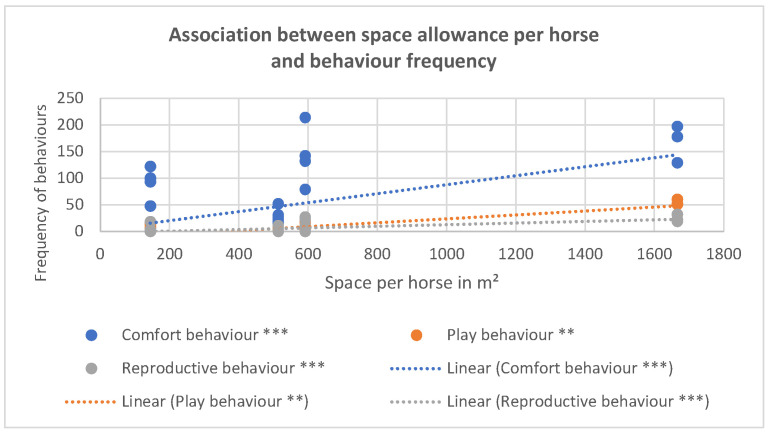
Association between space per horse and frequency of comfort, play and reproductive behaviours (Mann–Whitney U test: all *p* < 0.01); **: *p* ≤ 0.01; ***: *p* ≤ 0.001; the dotted trend lines represent a linear regression and illustrate the association between space per horse and these behaviours.

Stallions exhibited significantly higher levels of reproductive behaviour than geldings (Mann–Whitney U test: *N* = 35, W = 280.5, *p* < 0.001; Figure 3; Table 4 and Table 5). Group size and space availability per horse were positively associated with reproductive behaviour, with larger groups (GLM: *N* = 35, t = 4.65, *p* < 0.001) and greater space availability corresponded to higher levels of reproductive behaviour (GLM: *N* = 35, t = 4.83, *p* < 0.001; Figure 4). In contrast, age was negatively associated with reproductive behaviour, with older horses displaying lower levels of reproductive behaviour (GLM: *N* = 35, t = −3.11, *p* = 0.004).

#### 3.1.7. Comfort Behaviour

Comfort behaviour did not differ significantly between stallions and geldings (Mann–Whitney U test: *N* = 35, W = 198.5, *p* = 0.14; Table 4 and Table 5). Space availability per horse was positively associated with comfort behaviour, with greater space corresponding to higher levels of comfort behaviour (GLM: *N* = 35, t = 7.57, *p* < 0.001; Figure 4). Similarly, resting behaviour was positively associated with comfort behaviour (GLM: *N* = 35, t = 3.85, *p* < 0.001). Group size was negatively associated with comfort behaviour, with higher levels observed in smaller groups (GLM: *N* = 35, t = −4.87, *p* < 0.001). Age was not significantly associated with comfort behaviour (GLM: *N* = 35, *p* > 0.05).

#### 3.1.8. Avoidance Behaviour

Avoidance behaviour did not differ significantly between stallions and geldings (Mann–Whitney U test: *N* = 35, W = 114.5, *p* = 0.21; Table 4 and Table 5). Group size, age and space availability per horse (Figure 2) were significantly positively associated with avoidance behaviour. Group size was negatively associated with avoidance behaviour, with higher levels observed in smaller groups (GLM: *N* = 35, t = −3.47, *p* = 0.002). Age was also negatively associated with avoidance behaviour, with older horses displaying lower levels of avoidance behaviour (GLM: *N* = 35, t = −2.42, *p* = 0.02). In addition, space availability per horse was negatively associated with avoidance behaviour, indicating that horses with less space exhibited higher levels of avoidance behaviour (GLM: *N* = 35, t = −2.77, *p* = 0.01; Figure 2).

#### 3.1.9. Resting Behaviour

Resting behaviour did not differ significantly between stallions and geldings (Mann–Whitney U test: *N* = 35, W = 184.5, *p* = 0.31; Table 4 and Table 5). Group size was negatively associated with resting behaviour, with higher levels observed in smaller groups (GLM: *N* = 35, t = −8.93, *p* < 0.001). Age was also negatively associated with resting behaviour, with older horses displaying lower levels of resting behaviour (GLM: *N* = 35, t = −2.10, *p* = 0.04). In addition, space availability per horse was positively associated with resting behaviour (GLM: *N* = 35, t = 2.98, *p* = 0.01).

#### 3.1.10. Play Behaviour

**Figure 5:**
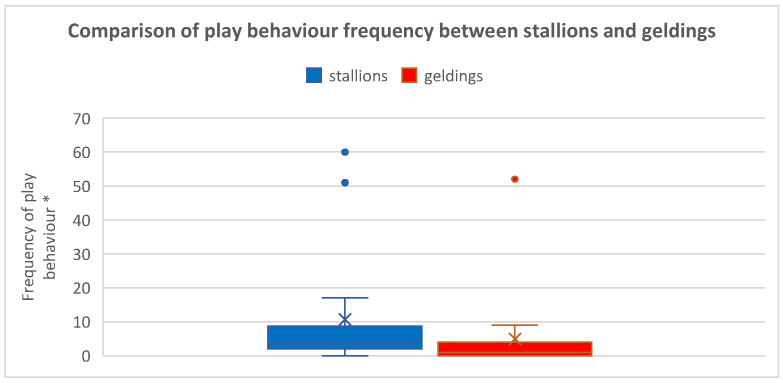
Stallions showed higher levels of play behaviour than geldings (Mann–Whitney U test: W = 225, *p* = 0.02). *: *p* ≤ 0.05.

Stallions engaged more frequently in play behaviour with conspecifics than geldings (Mann–Whitney U test: *N* = 35, W = 240, *p* < 0.001; Table 4 and Table 5). Furthermore, stallions also showed higher overall levels of play behaviour than geldings (Mann–Whitney U test *N* = 35, W = 225, *p* = 0.02; Figure 5; Table 4 and Table 5). The age of the horses had no effect on play behaviour (GLM: *N* = 35, *p* > 0.05). Space availability per horse was positively associated with play behaviour, with greater space corresponding to higher levels of play behaviour (GLM: *N* = 35, t = 3.49, *p* = 0.002; Figure 4).

### 3.2. Analysis of Movement

Horses covered a mean distance of 2.36 km during each eight-hour observation period (SD = 0.67). Space availability per horse was negatively correlated with average movement distance, indicating that horses with less space covered greater distances during the observation period (Spearman’s rank correlation: *N* = 15, r_s_ = −0.55, *p* = 0.03). Age and breed were not significantly associated with average movement distance (GLM: *N* = 15, *p* > 0.05). In addition, average movement distance did not differ significantly between stallions and geldings (Mann–Whitney U test: *N* = 35, W = 27, *p* = 1; Table 4 and Table 5). Ritualised behaviour was positively associated with greater movement distance (GLM: N = 15, t = 2.34, *p* = 0.04).

## 4. Discussion

The most important finding of this study is that no injuries were observed in stallions and geldings housed together throughout the entire observation period and that attack behaviour did not differ significantly between the stallions and geldings. This is in contrast with other studies, in which stallions have often been considered difficult to manage [5,28]. They are therefore frequently kept isolated due to their displays of sexual behaviour, their presumed higher level of aggression, and concerns regarding injury risks [5,28]. However, stallions generally display only the minimum level of aggression required in a given situation, and rarely sustain serious injuries [5,29], although play fighting can pose a certain risk of injury [5]. The present study found, similar frequencies of attack behaviour in stallions and geldings, and this is consistent with previous studies that showed that castration does not necessarily reduce aggression levels [22]. The present findings challenge the general assumption that stallions and geldings are incompatible in group housing and suggest that, under appropriate housing conditions, group housing of stallions and geldings does not necessarily increase aggression or the risk of injuries.

Moreover, in the present study, attack behaviour was the least frequently observed behavioural category suggesting that severe aggressive interactions were largely absent under the housing conditions investigated and may explain the absence of injuries in the mixed housing of stallions and geldings.

While age did not significantly influence attack behaviour in the present study, other authors found that young Przewalski’s horses showed lower levels of aggressiveness than adults under comparable conditions [30]. This discrepancy may be related to differences in subspecies, social structure, management, and environmental conditions. As attack behaviour occurred only rarely in the present study, this may have limited the ability to detect age-related differences.

A further key finding of this study is that stallions exhibited significantly higher levels of both affiliative and agonistic behaviour than geldings. This finding partly contrasts with the results of King et al. [22], who reported higher levels of affiliative behaviour in geldings following castration, whereas the frequency of agonistic behaviour remained unaffected [22].

Stallions exhibited significantly higher levels of reproductive behaviour than geldings in the present study, in line with the King et al., who reported a tendency for lower levels of reproductive behaviour in geldings compared with stallions, although this difference was not statistically significant [22]. In addition, stallions showed significantly higher levels of dominance behaviour than geldings. This pattern is also consistent with the findings of King et al. [22], who observed lower levels of marking behaviour in geldings, which can be interpreted as a reduced expression of dominance-related behaviours [22]. Furthermore, in line with earlier findings, stallions exhibited both more play behaviour [31] and more ritualised behaviour [32,33,34] than geldings.

Across all individuals, affiliative behaviours in this study were observed significantly less frequently than agonistic behaviours, as has been reported in certain contexts in feral horses [35]. This is also consistent with the findings of Briefer Freymond et al. [19], who observed higher frequencies of agonistic than affiliative interactions in stallions following group integration, with affiliative behaviours increasing over time as social relationships developed [19].

Agonistic interactions are a distinctive feature of bachelor bands [14]. Bachelor bands in the wild are typically composed of immature stallions, generally between 1 and 6 years of age, although older stallions may occasionally be present [12,16]. Accordingly, the stallion behaviours observed in the present study resembled that seen in feral horses’ bachelor bands [12], which may explain the higher occurrence of agonistic behaviour, particularly as the stallions of the present study included younger horses.

No stereotypic behaviour was observed in either geldings or stallions in this study. This contrasts previous findings, which report that stereotypic behaviours occur most frequently in stallions [5,36]. The reported elevated stereotypic behaviour in stallions may be caused by insufficient space and limited social contact, both of which compromise the expression of natural behaviour [5,36], and the stallions of the present study were kept with ample space and unlimited conditions for social interactions.

The present study indicates a positive association between space availability and the frequency of some social behaviours in horses [4]. Play, reproductive, resting, and comfort behaviours increased with increasing space. Horses may be more likely to exhibit increased resting and comfort behaviour in larger areas if they can find suitable resting areas that meet their safety and comfort needs [37].

However, with increasing space availability, dominance behaviours decreased significantly, and attacks tended to occur less frequently, indicating reduced levels of aggression. This is consistent with the recommendations of Flauger and Krueger [38], who suggest a space availability of at least 331 m^2^ per horse to reduce aggression and injury risk. Similarly, previous studies have reported that the frequency of agonistic behaviour is influenced by enclosure size, with higher levels of aggression observed in smaller enclosures [5,30].

In the present study, the horses moved an average distance of 2.36 km in eight hours. In comparison, other authors report daily movement distances from 5 to 16 km, depending on the husbandry system, terrain and survey method [39,40,41]. As the movement data in this study were recorded only over a limited period, it remains unclear to what extent activity differs over the entire day. For this reason, no extrapolation of the collected data to 24 h was made. Hildebrandt et al. [39] found no significant effect of sex on movement activity when comparing mares and geldings. The authors suggested that the absence of stallions may have contributed to this finding, because stallions frequently display agonistic and reproductive behaviours that are associated with increased movement activity [32]. However, despite the inclusion of stallions in the present study, average movement activity did not differ significantly between stallions and geldings. Furthermore, the proposed positive association between space availability and movement activity [39] could not be confirmed in the present study. One possible explanation is that movement data were collected over a period of only eight hours rather than 24 h.

The present study has several limitations that should be addressed in future research. A major limitation is the confounding factor of the relationship between the horses’ behaviour and their age. The stallions were considerably younger than the geldings. These factors cannot be separated statistically within the present dataset. Consequently, it is not possible to determine whether the observed differences in behavioural activity were due to castration, age, or a combination of both. Therefore, the findings should not be interpreted as describing behaviour alone, but rather as the combined effects of group composition. Accordingly, causal inferences regarding castration alone cannot be made based on the present data. Due to the limited sample size, uneven age distribution between groups and the structure of the dataset, additional age-stratified or covariate-based analyses were not feasible without compromising model stability.

GPS trackers were left on the horses during breaks in some cases, whilst they were removed in others. Although the break periods in question could be identified and corrected retrospectively, a consistent approach would have improved the analysis. Additionally, movement data were collected for only three consecutive days per horse and from a limited number of individuals, which restricts the generalisability of the results. External factors, such as weather conditions, may also have influenced movement patterns.

The sample size for the movement data was small, comprising 15 horses, due to the number of trackers available. The behavioural observations were conducted close to the horses, which may have influenced the horses’ behaviour [23]. At some facilities, it was not possible to observe the horses from outside the paddocks, as this would have prevented a view of the entire area and thus of all the horses. Consequently, the observers sometimes had to stand inside the paddocks, which may also have influenced the horses’ behaviour [23]. As behavioural observations and GPS measurements were conducted exclusively during daytime, the results of this study are limited in this respect. The observations were carried out between late July and mid-September, meaning that seasonal differences in the horses’ behaviour could not be recorded. Individual horses were occasionally removed from the group for rides or other activities, resulting in a reduced amount of data for these individuals.

Another methodological limitation relates to the convenience sampling of facilities. Although the premises were selected based on predefined inclusion criteria, the small number of eligible and available facilities meant that the sample size was limited. This ensured that all participating facilities met the study’s requirements and reflected the management practices of housing stallions and geldings in groups. However, this procedure may have limited the comparability of the different facilities with other establishments and reduced the generalisability of the findings to different management systems.

Furthermore, the use of a predefined behaviour classification table may have limited the detection of unexpected behaviours. However, no such behaviours were observed during this study.

## 5. Conclusions

This study found no significant difference in attack behaviour between stallions and geldings. Furthermore, no injuries or stereotypic behaviours were observed throughout the entire observation period. In general, more affiliative, agonistic, ritualised, reproductive, dominance and play behaviours were observed in stallions than in geldings. By contrast, there was no significant difference between stallions and geldings in the occurrence of comfort, resting, threatening and avoidance behaviours. Management factors, particularly group size and space availability, were associated with several behavioural parameters.

Despite these behavioural differences, no significant difference in overall movement activity was detected between stallions and geldings.

Further research is needed, particularly regarding the effects of group housing on stress levels and social hierarchies and GPS measurements over 24 h periods to establish whether movement activity differs between stallions and geldings when nighttime activity is taken into account.

Overall, the findings indicate that mixed group housing of stallions and geldings is feasible under appropriate management and housing conditions.

## Figures and Tables

**Table 2:** Overview of the horses at the different equestrian facilities.

Equestrian Facility	Identification Number	Reproductive Status	Breed	Age
1	1	Stallion	Tinker	9
	2	Gelding	Lusitano mix	14
	3	Gelding	Lusitano mix	17
	4	Gelding	Noriker	3
	5	Stallion	Lusitano	1
	6	Gelding	Westphalian	18
	7	Gelding	Andalusian	12
	8	Stallion	Noriker	7
	9	Gelding	Welsh pony mix	17
	10	Gelding	Arabian	11
	11	Stallion	Noriker	7
	12	Gelding	French Trotter	18
	13	Gelding	Mixed breed	19
	14	Stallion	Hucul pony	11
	15	Stallion	Noriker	6
	16	Stallion	Noriker	9
	17	Gelding	Mixed breed	11
	18	Stallion	Lusitano mix	13
	19	Gelding	Mixed breed	10
	20	Stallion	Quarter horse	4
	21	Stallion	Quarter horse	3
	22	Stallion	Quarter horse	1
	23	Stallion	Mixed breed	1
	24	Gelding	Mixed breed	12
2	25	Stallion	Marismeño horse	12
	26	Stallion	Andalusian	14
	27	Gelding	Shetland pony	18
	28	Gelding	Shetland pony	18
3	29	Stallion	Arabian, Barb horse, PRE, Lusitano mix	11
	30	Stallion	Shetland pony	16
	31	Stallion	Black Forest Draught Horse	3
	32	Gelding	Garrano, PRE mix	17
4	33	Stallion	Arabian	17
	34	Stallion	PRE	7
	35	Gelding	Pony mix	15

**Table 3:** Classification of horse behaviours.

Behavioural Category	Behaviours
Attack behaviour	Striking, kicking, biting, displacing, aggressive approach
Threat behaviour	Threatening postures and actions including pinned ears, snaking and biting
Dominance behaviour	Overmarking with urine/faeces, sniffing faeces, chasing, herding
Ritualised behaviour	Foreleg striking, neck arching, squealing, head tossing
Reproductive behaviour	Mounting, flehming, extending the penis, masturbation
Comfort behaviour	Approach for grooming, grooming, sniffing, snorting, rolling, shaking, fly swishing
Avoidance behaviour	Retreating, moving away, flight behaviour
Resting behaviour	Resting while standing, sleeping while standing, yawning, stretching, recumbent resting
Play behaviour	Social playing (pinching, rage, etc.), running, play fighting (rearing, kicking, kneeling, circling)
Submissive behaviour	Kneeling, foal snapping
Social behaviour	Whinnying, sniffing faeces, following, approaching
Elimination behaviour	Defecation, urination
Shelter and comfort seeking	Seeking shelter, turning backs to weather as a natural windbreak, sunning
Ingestion behaviour	Grazing, recumbent grazing, browsing, drinking
Orientation behaviour	Distant orientation, close-range orientation, sniffing
Locomotion behaviour	Standing alert, walking, trotting, cantering, jumping, stampeding

**Table 4:** Frequencies of behavioural parameters and movement activity (mean distance in kilometres per eight ours observation period) in stallions and geldings (*N* = 35, GPS activity: *n* = 15).

Parameter	Stallions (Median)	Min.	Max.	Geldings (Median)	Min.	Max.
Affiliative behaviour	65	14	236	31	9	60
Agonistic behaviour	74	22	169	43	19	65
Attack behaviour	4	0	50	2	0	13
Threatening behaviour	22.5	0	81	11	2	36
Dominance behaviour	21.5	3	86	1	0	7
Ritualised behaviour	19.5	0	43	0	0	4
Reproductive behaviour	6.5	0	32	0	0	22
Comfort behaviour	17	0	54	11	0	62
Avoidance behaviour	16.5	2	53	21	7	42
Resting behaviour	18	5	54	15	4	62
Play behaviour	4	0	60	1	0	52
Movement activity	2.23	-	-	2.31	-	-

**Table 5:** Comparison of the median frequencies of behavioural parameters and movement activity between stallions and geldings (Mann–Whitney U tests) across the total observation period of 96 h.

Parameter	Statistic	*p*-Value	Higher Frequency Observed in Stallions/Geldings
Affiliative behaviour	W = 227	0.02 *	Stallions
Agonistic behaviour	W = 254.5	<0.001 ***	Stallions
Attack behaviour	W = 174	0.49 n.s.	-
Threatening behaviour	W = 205	0.09 n.s.	Stallions
Dominance behaviour	W = 290	<0.001 ***	Stallions
Ritualised behaviour	W = 261.5	<0.001 ***	Stallions
Reproductive behaviour	W = 280.5	<0.001 ***	Stallions
Comfort behaviour	W = 198.5	0.14 n.s.	-
Avoidance behaviour	W = 114.5	0.21 n.s.	-
Resting behaviour	W = 184.5	0.31 n.s.	-
Play behaviour	W = 225	0.02 *	Stallions
Movement activity	W = 27	1 n.s.	-

n.s. = not significant; *: *p* ≤ 0.05; ***: *p* ≤ 0.001.

## Data Availability

The original contributions presented in the study are included in the article; further inquiries can be directed to the corresponding authors.

## Supplementary Materials

The following supporting information can be downloaded at: https://www.mdpi.com/article/10.3390/vetsci13070660/s1, File S1: Statistics; Table S1: Raw Data.
