# Supplementary material for: Behaviour and Movement Activity of Stallions and Geldings in Group Housing
Source: Vet Sci. 2026 Jul 7;13(7):660. doi: 10.3390/vetsci13070660 (PMC13417708; doi:10.3390/vetsci13070660)
Supplement: Supplementary file 1 [file vetsci-13-00660-s001.zip › vetsci-4393614-supplementary.pdf]

File S1

Behaviour and Movement Activity of Stallions and Geldings in Group Housing

Rhoda C. Apitzsch, Sarah Handel and Konstanze Krueger *

Faculty of Agriculture, Economics and Management, Nuertingen-Geislingen University, Neckarsteige 6-10, 72622 Nuertingen, Germany; r.apitzsch@mqs.de, sarah@tssl-handel.de

* Correspondence: konstanze.krueger@hfwu.de

Appendix B: R-Outputs

Binomial tests:

Affiliative and agonistic behaviour:

Data: c(1968, 2141)

Number of successes = 1968, number of trials = 4109, p-value = 0.007284

Mann-Whitney U tests:

Affiliative behaviour:

Rcmdr+ data=Dataset) # medians by group

stallions geldings

65 31

Wilcoxon rank sum test with continuity correction

data: affiliative by reproductive.status...4

W = 227, p-value = 0.01524

Agonistic behaviour:

Rcmdr+ data=Dataset) # medians by group

stallions geldings

74 43

Wilcoxon rank sum test with continuity correction

data: agonistic by reproductive.status...4

W = 254.5, p-value = 0.0008569

Attack behaviour:

Rcmdr+ data=Dataset) # medians by group

stallions geldings

4 2

Wilcoxon rank sum test with continuity correction

data: attack by reproductive.status...4

W = 174, p-value = 0.4953

Threatening behaviour:

Rcmdr+ data=Dataset) # medians by group

stallions geldings

22.5 11.0

Wilcoxon rank sum test with continuity correction

data: threatening by reproductive.status...4

W = 205, p-value = 0.08889

Dominance behaviour:

Rcmdr+ data=Dataset) # medians by group

stallions geldings

21.5 1.0

Wilcoxon rank sum test with continuity correction

data: dominance by reproductive.status...4

W = 290, p-value = 0.000006007

Ritualised behaviour:

Rcmdr+ # medians by group

stallions geldings

19.5 0.0

Wilcoxon rank sum test with continuity correction

data: ritualised by reproductive.status...4

W = 261.5, p-value = 0.0002256

Reproductive behaviour:

Rcmdr+ # medians by group

stallions geldings

6.5 0.0

Wilcoxon rank sum test with continuity correction

data: reproductive by reproductive.status...4

W = 280.5, p-value = 0.00001446

Comfort behaviour:

Rcmdr+ # medians by group

stallions geldings

26 15

Wilcoxon rank sum test with continuity correction

data: comfort by reproductive.status...4

W = 198.5, p-value = 0.1373

Avoidance behaviour:

Rcmdr+ # medians by group

stallions geldings

16.5 21.0

Wilcoxon rank sum test with continuity correction

data: avoidance by reproductive.status...4

W = 114.5, p-value = 0.2095

Resting behaviour:

Rcmdr+ # medians by group

stallions geldings

18 15

Wilcoxon rank sum test with continuity correction

data: resting by reproductive.status...4

W = 184.5, p-value = 0.3056

Play behaviour within-status interactions:

Rcmdr+ medians by group

stallions geldings

2.5 0.0

Wilcoxon rank sum test with continuity correction

data: play within-status interactions by reproductive.status...4

W = 240, p-value = 0.00008753

Play behaviour:

Rcmdr+ medians by group

stallions geldings

4 1

Wilcoxon rank sum test with continuity correction

data: play by reproductive.status...4

W = 225, p-value = 0.01724

Analysis of Movement:

Rcmdr+ data=Dataset) # medians by group

stallions geldings

2,23 2,305

Wilcoxon rank sum exact test

data: movement by reproductive.status...3

W = 27, p-value = 1

GLM:

Affiliative behaviour:

Call: glm(formula = affiliative ~ space.horse + age, family = quasipois-son(log), data = Dataset)

Coefficients:

Estimate Std. Error t value Pr(>|t|)

(Intercept) 3.5391621 0.3951138 8.957 4.15e-10 ***

space.horse 0.0001999 0.0003572 0.560 0.57978

age -0.0239803 0.0280988 -0.853 0.39997

Agonistic behaviour:

Call: glm(formula = agonistic~ equestrian.facility + reproductive.status + group.size + housing + attack + age, family = quasipoisson(log), data = Dataset)

Coefficients:

Estimate Std. Error t value Pr(>|t|)

(Intercept) 5.272974 0.477623 11.040 1.04e-11 ***

equestrian.facility -0.536648 0.358413 -1.497 0.14551

reproductive.status -0.473377 0.165445 -2.861 0.00789 **

group.size -0.029494 0.015118 -1.951 0.06113 .

housing 0.328742 0.299688 1.097 0.28201

attack 0.026502 0.004386 6.043 1.63e-06 ***

age 0.005774 0.014220 0.406 0.68780

Attack behaviour:

Call: glm(formula = attack ~ equestrian.facility + age + reproductive.status + group.size + housing, family = quasipoisson(log), data = Dataset)

Coefficients:

Estimate Std. Error t value Pr(>|t|) 68

(Intercept) 1.38967 4.06431 0.342 0.7349

equestrian.facility -2.68888 3.16824 -0.849 0.4030

age 0.07410 0.05625 1.317 0.1980

reproductive.status -1.17842 0.63638 -1.852 0.0743 .

group.size 0.09128 0.15381 0.593 0.5575

housing 1.97374 3.00789 0.656 0.5169

Call: glm(formula = attack ~ space.horse + ritualised + threatening + dominance, family = quasipoisson(log), data = Dataset)

Coefficients:

Estimate Std. Error t value Pr(>|t|)

(Intercept) 2.264504 0.436037 5.193 0.0000148 ***

space.horse -0.001114 0.000602 -1.851 0.07438 .

ritualised -0.008746 0.012638 -0.692 0.49441

threatening 0.038160 0.013980 2.730 0.01067 *

dominance 0.016335 0.006007 2.719 0.01093 *

Dominance behaviour:

Call: glm(formula = dominance ~ equestrian.facility + age + housing, family = quasipoisson(log), data = Dataset)

Coefficients:

Estimate Std. Error t value Pr(>|t|)

(Intercept) 2.86455 1.25044 2.291 0.02943 *

equestrian.facility -4.92411 1.73816 -2.833 0.00831 **

age 0.08920 0.03227 2.764 0.00981 **

housing 3.47769 1.20554 2.885 0.00732 **

Call: glm(formula = dominance~ space.horse + reproductive, family = quasipoisson(log), data = Dataset)

Coefficients:

Estimate Std. Error t value Pr(>|t|)

(Intercept) 3.9709387 0.5913724 6.715 0.000000193 ***

space.horse -0.0029857 0.0008202 -3.640 0.001016 **

reproductive 0.1685930 0.0384355 4.386 0.000131 ***

Ritualised behaviour:

Call: glm(formula = ritualised ~ reproductive.status + age, family = quasipoisson(log), data = Dataset)

Coefficients:

Estimate Std. Error t value Pr(>|t|)

(Intercept) 5.46664 0.71088 7.690 0.00000000913 ***

reproductive.status -3.57424 0.67115 -5.326 0.00000770585 ***

age 0.09497 0.02739 3.467 0.00152 **

Call: glm(formula = ritualised ~ reproductive + movement + space.horse, family = quasipoisson(log), data = Dataset)

Coefficients:

Estimate Std. Error t value Pr(>|t|)

(Intercept) 0.24279 0.85926 0.283 0.7823

reproductive 0.05533 0.02186 2.531 0.0264 *

movement 0.60811 0.25953 2.343 0.0372 *

space.horse -0.0004182 0.0031899 -0.131 0.899

Reproductive behaviour:

Call: glm(formula = reproductive ~ group.size + age + housing + dominance, family = quasipoisson(log), data = Dataset)

Coefficients:

Estimate Std. Error t value Pr(>|t|)

(Intercept) -3.587249 1.131106 -3.171 0.003569 **

group.size 0.304041 0.065449 4.645 0.0000678918 ***

age -0.064089 0.020588 -3.113 0.004141 **

housing 2.294521 0.377102 6.085 0.0000012632 ***

dominance 0.031417 0.004199 7.481 0.0000000303 ***

Call: glm(formula = reproductive ~ space.horse, family = quasipoisson(log), data = Dataset)

Coefficients:

Estimate Std. Error t value Pr(>|t|)

(Intercept) -0.1133373 0.4855132 -0.233 0.81696

space.horse 0.0014675 0.0003037 4.833 0.0000347 ***

Comfort behaviour:

Call: glm(formula = comfort ~ housing + group.size, family = quasipoisson(log), data = Dataset)

Coefficients:

Estimate Std. Error t value Pr(>|t|)

(Intercept) 4.37811 0.39731 11.020 2.00e-12 ***

housing 0.19910 0.08504 2.341 0.0256 *

group.size -0.07448 0.01531 -4.866 2.94e-05 ***

Call: glm(formula = comfort ~ space.horse + age + resting, family = quasipoisson(log), data = Dataset)

Coefficients:

Estimate Std. Error t value Pr(>|t|)

(Intercept) 1.6013026 0.2417604 6.624 0.0000002925 ***

space.horse 0.0010927 0.0001441 7.586 0.0000000231 ***

age -0.0130789 0.0162002 -0.807 0.42605

resting 0.0242184 0.0065095 3.720 0.00085 ***

Avoidance behaviour:

Call: glm(formula = avoidance ~ group.size + age + space.horse, family = quasipoisson(log), data = Dataset)

Coefficients:

Estimate Std. Error t value Pr(>|t|)

(Intercept) 3.6360340 0.3626972 10.025 4.32e-11 ***

group.size -0.0226329 0.0106714 -2.121 0.04231 *

age -0.0508366 0.0214446 -2.371 0.02438 *

space.horse -0.0008766 0.0003167 -2.767 0.00959 **

Resting behaviour:

Call: glm(formula = resting~ group.size + age + equestrian.facility + comfort + reproductive.status + space.horse, family = quasipoisson(log), data = Dataset)

Coefficients:

Estimate Std. Error t value Pr(>|t|)

(Intercept) 4.751170 0.280727 16.925 < 2e-16 ***

group.size -0.076404 0.008510 -8.978 7.18e-10 ***

age -0.025287 0.010532 -2.401 0.02298 *

equestrian.facility -0.312017 0.089856 -3.472 0.00164 **

comfort 0.002941 0.001356 2.168 0.03847 *

reproductive.status 0.124032 0.104346 1.189 0.24422

space.horse 0.003396 0.001003 2.98 0.00912 **

Play behaviour:

Call: glm(formula = play ~ group.size + equestrian.facility + space.horse + housing, family = quasipoisson(log), data = Dataset)

Coefficients:

Estimate Std. Error t value Pr(>|t|)

(Intercept) 4.2209591 2.1295770 1.982 0.05670 .

group.size -0.1339335 0.0759544 -1.763 0.08803 .

equestrian.facility -3.8360445 1.4735244 -2.603 0.01422 *

space.horse 0.0034758 0.0009952 3.492 0.00151 **

housing 1.9459101 0.9772006 1.991 0.05562 .

Call: glm(formula = play ~ group.size + age + equestrian.facility + space.horse + housing, family = quasipoisson(log), data = Dataset)

Coefficients:

Estimate Std. Error t value Pr(>|t|)

(Intercept) 4.445094 2.199843 2.021 0.05297 .

group.size -0.131560 0.077343 -1.701 0.10003

age 0.004949 0.022778 0.217 0.82958

equestrian.facility -3.502776 1.506065 -2.326 0.02750 *

space.horse 0.003396 0.001003 3.386 0.00212 **

housing 1.713825 1.002713 1.709 0.09848 .

<u>Movement:</u>

glm(formula = movement ~ housing + equestrian.facility + age, family = Gamma(log), data = Dataset)

Coefficients:

Estimate Std. Error t value Pr(>|t|)

(Intercept) 3.472199 0.636497 5.455 0.000279 ***

housing 0.399963 0.174568 2.291 0.044925 *

equestrian.facility -1.611079 0.408678 -3.942 0.002765 **

age -0.0139130 0.0314111 -0.443 0.668268

Call: glm(formula = ritualised ~ reproductive + movement, family = quasipoisson(log), data = Dataset)

Coefficients:

Estimate Std. Error t value Pr(>|t|)

(Intercept) 0.24279 0.85926 0.283 0.7823

reproductive 0.05533 0.02186 2.531 0.0264 *

movement 0.60811 0.25953 2.343 0.0372 *

Mean:

Rcmdr> numSummary(Dataset[,"movement", drop=FALSE], statistics=c("mean",

Rcmdr+ "sd", "IQR", "quantiles"), quantiles=c(0,.25,.5,.75,1))

mean sd IQR 0% 25% 50% 75% 100% n NA

2.363022 0.667041 0.6378616 1.55 1.992138 2.28 2.63 4.181053 15 20

Spearman's rank correlation rho

data: space.horse and movement

S = 744, p-value = 0.03006

alternative hypothesis: true rho is not equal to 0

sample estimates:

rho

-0.5503673

Table S1: Raw Data

Behaviour and Movement Activity of Stallions and Geldings in Group Housing

Rhoda C. Apitzsch, Sarah Handel, Konstanze Krueger

animal item	equestrian.facility	reproduction.status	reproductive	age	space.horse	housing	movement
1	1	1	stallion	9	513	1	2,75
2	1	2	gelding	14	513	1	
3	1	2	gelding	17	513	1	
4	1	2	gelding	3	513	1	
5	1	1	stallion	1	513	1	
6	1	2	gelding	18	513	1	
7	1	2	gelding	12	513	1	2,35
8	1	1	stallion	7	513	1	2,14
9	1	2	gelding	17	513	1	
10	1	2	gelding	11	513	1	
11	1	1	stallion	7	513	1	
12	1	2	gelding	18	513	1	
13	1	2	gelding	19	513	1	
14	1	2	gelding	11	513	1	
15	1	1	stallion	6	513	1	
16	1	1	stallion	9	513	1	
17	1	2	gelding	11	513	1	2,33
18	1	1	stallion	13	513	1	
19	1	2	gelding	10	513	1	
20	1	1	stallion	4	513	1	
21	1	1	stallion	3	513	1	
22	1	1	stallion	1	513	1	
23	1	1	stallion	1	513	1	
24	1	2	gelding	12	513	1	
25	2	1	stallion	12	144	3	4,18
26	2	1	stallion	14	144	3	3,07
27	2	2	gelding	18	144	2	2,79
28	2	2	gelding	18	144	2	2,07
29	3	1	stallion	11	591	4	1,55
30	3	1	stallion	16	591	4	1,61
31	3	1	stallion	3	591	4	1,91
32	3	2	gelding	17	591	4	2,28
33	4	1	stallion	17	1666	5	2,23
34	4	1	stallion	7	1666	5	2,51
35	4	2	gelding	15	1666	5	1,67

group .size	attack within-status interactions	attack between-status interactions	attack	ritualised within-status interactions	ritualised between-status interact
32	18	50	7	0	
24	4	7	11	0	0
24	1	0	1	0	0
24	1	2	3	0	0
24	1	0	1	0	0
24	1	1	2	0	0
24	8	5	13	0	1
24	1	11	12	19	0
24	0	6	6	0	0
24	0	2	2	0	0
24	0	2	2	23	0
24	3	3	6	0	2
24	1	0	1	0	0
24	2	0	2	0	0
24	5	3	8	22	0
24	1	4	5	20	0
24	1	4	5	0	1
24	3	2	5	16	0
24	0	6	6	0	0
24	3	2	5	7	0
24	2	1	3	1	0
24	1	2	3	0	0
24	0	0	0	0	0
24	2	0	2	0	3
4	5	6	11	28	14
4	0	5	5	22	0
4	0	0	0	0	0
4	0	1	1	0	2
4	6	2	8	17	0
4	1	0	1	19	1
4	0	0	0	0	0
4		3	3		0
3	1	0	1	8	4
3	3	0	3	15	7
3		0	0		0

ritualised nobody	rituali sed	dominance within-status interactions	dominance between-status interactions	dominance nobody
0	7	8	18	60
0	0	0	0	0
0	0	0	0	1
0	0	0	0	4
0	0	1	0	2
0	0	0	0	0
0	1	0	0	0
5	24	1	3	21
0	0	1	0	3
0	0	0	0	0
2	25	0	1	10
0	2	0	0	0
0	0	0	0	0
0	0	0	0	1
2	24	1	0	16
3	23	0	0	20
2	3	0	0	7
2	18	0	0	41
0	0	0	0	6
0	7	0	0	23
0	1	0	0	4
1	1	0	0	3
0	0	0	0	4
1	4	0	0	1
1	43	1	3	44
1	23	0	0	27
0	0	0	0	1
0	2	0	0	3
4	21	1	2	64
4	24	3	0	75
0	0	1	0	40
0	0		0	3
3	15	6	1	11
4	26	5	0	7
1	1		1	1

dominance	play within-status interactions	play between-status interactions	play	comfort within-status interactions	comfort between-status interactions
86	2	1	3	0	2
0	0	0	0	1	2
1	2	0	2	0	0
4	0	1	1	1	8
3	1	0	1	0	8
0	0	0	0	3	1
0	0	0	0	1	5
25	0	0	0	0	6
4	2	1	3	1	1
0	0	3	3	4	3
11	2	1	3	4	9
0	0	0	0	2	0
0	1	0	1	1	0
1	0	0	0	0	2
17	6	1	7	4	1
20	3	0	3	2	3
7	0	5	5	3	0
41	4	2	6	0	1
6	0	0	0	0	2
23	6	1	7	1	2
4	4	1	5	2	0
3	0	1	1	0	0
4	1	2	3	0	1
1	0	5	5	0	0
48	11	3	14	0	0
27	2	5	7	0	1
1	1	0	1	1	0
3	0	2	2	0	0
67	2	0	2	1	0
78	11	6	17	2	8
41	2	0	2	1	12
3		9	9		18
18	31	20	51	0	0
12	30	30	60	0	1
2		52	52		1

comfort nobody	comfort	resting	feeding within-status interactions	feeding between-status interactions	threatening within-status interactions	threatening between-status int
20	22	14	20	1	18	22
8	11	15	0	15	1	13
5	5	11	0	16	2	0
18	27	7	0	11	1	3
9	17	18	21	26	0	0
7	11	4	0	4	5	11
12	18	16	0	6	14	12
7	13	17	9	5	8	25
6	8	14	0	4	2	0
3	10	15	0	7	5	4
4	17	14	8	17	2	1
4	6	11	0	5	3	2
5	6	13	0	8	6	0
2	4	13	0	5	7	4
25	30	5	6	2	22	9
26	31	12	3	4	2	6
26	29	19	0	2	4	22
11	12	11	8	10	11	5
50	52	20	0	6	3	8
5	8	12	7	0	9	11
11	13	15	8	2	6	4
10	10	19	11	3	1	1
14	15	18	19	22	0	0
15	15	12	0	5	1	6
122	122	53	4	37	20	19
47	48	54	4	9	11	30
99	100	50	22	9	1	7
93	93	40	22	37	1	12
131	132	34	107	32	57	24
132	142	30	97	33	15	3
66	79	47	79	41	20	5
196	214	62		111		16
197	197	37	34	10	25	7
177	178	43	34	24	21	15
128	129	33		34		36

threatening	avoidance within-status interactions	avoidance between-status interactions	avoidance	reproductive nobody	reproductive within-status interactions	reproductive between-status
40	5	1	6	9	1	1
14	4	15	19	0	0	0
2	2	14	16	0	0	0
4	9	13	22	1	0	0
0	13	5	18	0	0	0
16	2	16	18	0	0	0
26	2	23	25	0	0	0
33	6	2	8	6	0	0
2	0	11	11	0	0	0
9	9	22	31	0	0	0
3	12	1	13	4	0	1
5	4	22	26	0	0	0
6	2	15	17	0	0	0
11	5	12	17	2	0	0
31	20	3	23	5	0	0
8	14	3	17	5	0	0
26	5	16	21	1	0	0
16	14	1	15	8	0	0
11	3	24	27	0	0	0
20	26	11	37	6	1	0
10	26	5	31	2	0	0
2	24	2	26	3	0	0
0	15	9	24	5	0	0
7	4	3	7	0	0	0
39	2	0	2	17	0	1
41	53	0	53	2	1	0
8	0	34	34	0	0	0
13	1	37	38	0	0	0
81	9	0	9	22	0	0
18	1	1	2	27	0	0
25	29	4	33	11	0	0
16		42	42	0		0
32	3	0	3	19	0	0
36	13	3	16	32	0	0
36		9	9	22		0

reproductive	agonistic within-status interactions	agonistic between-status interactions	agonostic nobody	agonistic	affiliative within-status interactions
11	71	71	27	169	54
0	9	35	0	44	21
0	5	14	0	19	10
1	12	18	2	32	5
0	16	5	1	22	19
0	8	28	0	36	12
0	24	41	0	65	7
6	35	51	14	100	16
0	3	17	1	21	22
0	15	28	0	43	24
5	37	8	5	50	19
0	10	29	0	39	17
0	9	15	0	24	20
2	14	16	0	30	11
5	71	19	8	98	28
5	37	14	8	59	9
1	11	43	2	56	8
8	44	15	16	75	9
0	6	42	2	50	4
7	47	24	11	82	18
2	36	11	0	47	21
3	27	5	1	33	11
5	16	9	2	27	8
0	7	12	1	20	2
18	61	45	19	125	55
3	86	37	12	135	16
0	4	41	0	45	30
0	2	52	1	55	7
22	92	29	21	142	119
27	39	5	29	73	88
11	50	9	10	69	34
0		62	1	63	
19	43	12	5	60	70
32	57	25	5	87	44
22		45	1	46	

affiliative between-status interactions	affiliative nobody	affiliative
74	14	142
25	0	46
21	0	31
28	0	33
21	0	40
9	0	21
22	0	29
74	5	95
10	0	32
14	0	38
45	11	75
12	0	29
11	0	31
10	1	22
4	23	55
24	2	35
7	0	15
17	0	26
12	0	16
8	2	28
6	0	27
3	0	14
10	0	18
7	0	9
178	3	236
36	0	52
16	0	46
27	0	34
36	0	155
53	5	146
58	2	94
60	0	60
25	0	95
42	1	87
56	0	56
